# 

**Published:** 1898-04-30

**Authors:** 


**Notices of Births, Deaths, and Marriages** are inserted in this column at a charge of 2s. 6d. for an announcement not exceeding four lines.

### NOTICE TO CORRESPONDENTS.

All MS., Letters, Books for Review, and other matters intended for the Editor should be addressed The Editor, "The Hospital," The Hospital Building, 28 & 29, Southampton Street, Strand, W.C.

The Editor cannot undertake to return rejected MS., even when accompanied by stamped directed envelope.

**Notice to Advertisers and Subscribers.**

All Advertisements, Orders for Copies of The Hospital, and Business Communications should be addressed to **THE MANAGER, (Not to The Editor),** The Hospital, The Hospital Building, 28 & 29, Southampton Street, Strand, London, W.C.

The Cost of Subscription, post free, is as follows:—Per week, 2½d. per quarter, 2s. 8d.; per half-year, 5s. 3d.; per annum, 10s. 6d. Foreign:—Per annum, 15s. 2d.; payable, in all cases, in advance.

**NOTICE.—Vol. XXIII. of "The Hospital" (October, 1897, to March, 1898), in handsome cloth binding, will shortly be on sale at the Office of this Journal, price 7s. 6d. Cases for binding the Numbers contained in this Volume, 1s. 6d. Index to Vol. XXIII., 2½d., post free.**

**The Monthly Part for May will be ready on May 2nd. Price 6d., by post 8d.**

### Scraps and Gleanings.

It is proposed to establish a second dispensary in connection with the Glasgow Western Infirmary.

Over a thousand patients were treated at the Clinical Hospital for Women and Children, Manchester, in 1897.

The board of management of the British Home for Incurables have received a donation of 20 guineas from the Worshipful Company of Skinners.

The temporary isolation hospital erected by the Foleshill Rural District Council has now been completed. It is constructed to accommodate 12 patients.

The receipts of the Glasgow Hospital Sunday Fund show a slight decrease in 1897, as compared with 1896, the collections being £4,239 and £4,343 respectively.

The total receipts at the Dawlish Cottage Hospital during the past year amounted to £389, and the expenditure to £395. 67 in-patients were treated, 10 more than in 1896, and 134 out-patients received attention.

The Cottage Hospital, Warminster, has been the recipient of a gift of £5,000, £1,000 of which is to be used to build an additional ward, towards the endowment of which the remaining £4,000 is to be invested.

The financial statement submitted at the annual meeting of the Rochdale Infirmary shows that at the end of 1897 the debit balance had increased from £7 16s. to £216 3s. 264 in-patients and 1,873 out-patients were treated last year.

In consequence of the remodelling of the internal arrangements of the in-patient department of the Manchester Hospital for Consumption, the number of patients treated during 1897 decreased. The cost per patient was 17s. 2d. in 1897 against 16s. 4d. in 1896.

The Committee of the Royal Alexandra Hospital, Rhyl, have decided on the contract for the new hospital. The cost will be about £15,700 for the hospital block and £12,000 for the administrative block. As funds are not sufficient to allow the committee to at once proceed with the proposed rebuilding scheme in its entirety, the former block is to be put in hand first of all.

